# Stakeholders’ Views about the Management of Stable Chronic Conditions in Community Pharmacies

**DOI:** 10.3390/pharmacy10030059

**Published:** 2022-06-02

**Authors:** Mansour M. Alotaibi, Louise Hughes, Jenna L. Bowen, William R. Ford

**Affiliations:** 1 Pharmacy Practice Department, College of Clinical Pharmacy, King Faisal University, Al-Ahsa 31982, Saudi Arabia; mmqalotaibi@kfu.edu.sa; 2 School of Pharmacy and Pharmaceutical Sciences, Cardiff University, Cardiff CF10 3NB, UK; hughesml@cardiff.ac.uk (L.H.); bowenjl2@cardiff.ac.uk (J.L.B.)

**Keywords:** chronic conditions management, community pharmacy, community setting, long-term conditions’ management, chronic diseases management, managing chronic conditions, community pharmacists

## Abstract

The role of the community pharmacist has evolved to include the provision of more clinical services for patients. Those people who have stable chronic conditions will be managed in community pharmacies. This qualitative study used semi-structured in-depth interviews to understand the potential of providing additional patient-centred care for patients with stable chronic conditions in community pharmacies and identify potential limitations of this approach. Participants were recruited from Welsh Government, Local Health Boards (LHBS), Community Pharmacy Wales (CPW) and the Royal Pharmaceutical Society Wales (RPSW). The interviews were audio-recorded, transcribed verbatim, and analysed thematically. Eight interviews were conducted. The identified themes were as follows: (1) inconsistency and bureaucracy in commissioning pharmacy services; (2) availability of funding and resources; (3) disagreement and uncertainty about the contribution of the community pharmacy sector; (4) continuity of patient medical information and fragmented care; (5) accessibility, capacity and facilities in community pharmacy; (6) pharmacy education and clinical expertise, and (7) patient acceptability. It was clear that the potential benefit of managing stable chronic diseases in community pharmacies was recognised; however, several limitations expressed by stakeholders of pharmacy services need to be considered prior to moving forward.

## 1. Introduction

Managing stable chronic health conditions places a significant burden on the UK National Health Service (NHS). This has led to difficulties in accessing GPs and has a negative effect on public satisfaction with accessing health care [1]. An ageing population, plus a shortage in the number of GPs across the country, exacerbates the situation [2]; consequently, the NHS has to change to avoid collapse [3]. In response to this crisis, the Welsh Government (WG) started to fundamentally change how health care would be provided for patients. The WG recently launched a new vision and objectives toward 2030 in respect of having a healthier population [4,5]. The role of the community pharmacist has already evolved to include the provision of more clinical services for patients [6]; they are currently involved in providing more direct patient care, such as influenza vaccinations and discharge medicines review [7]; however, community pharmacists are currently not significantly involved in the clinical management of patients with stable chronic health conditions. According to the Welsh Pharmaceutical Committee, community pharmacists will be more involved in managing stable chronic health conditions by becoming independent prescribers [5]; they will be integrated with other health care providers at regional and local levels so that health services can be provided seamlessly; indeed, the way in which care is delivered will also change, allowing for more collaboration between pharmacies and other healthcare professionals. The focus of community pharmacists will be on the optimisation of therapeutic outcomes and prescribing, and therefore, every community pharmacy will have at least one pharmacist who is qualified as an independent prescriber (IP). More importantly, all people who have stable chronic conditions will be managed in community pharmacies [5]; this is projected to decrease demands on other NHS sectors such as GPs and may also improve patient access to healthcare services. To better understand the future involvement of community pharmacies in clinically managing stable chronic conditions, there was a need to capture opinions from the stakeholders of pharmacy services in Wales.

### The Aim of the Study

The aim of this study was to elicit the views of community pharmacy service stakeholders who are directly/indirectly involved in commissioning community pharmacy services in Wales; this included the Welsh Government, Local Health Boards (LHBs, seven health boards that are responsible for planning, securing and delivering healthcare services in Wales), Welsh Pharmaceutical Committee (WPC, a national committee that advises on pharmacy and the pharmaceutical profession), Community Pharmacy Wales (CPW, a professional body that represents all community pharmacy owners in Wales on NHS matters) and the Royal Pharmaceutical Society Wales (RPSW, a professional body for pharmacists in Wales), on the future of community pharmacy in clinically managing stable chronic conditions. The research objectives were to understand the potential of providing additional patient-centred care for patients with stable chronic conditions in community pharmacies and identify potential limitations of this approach.

## 2. Materials and Methods

The interpretivist paradigm was used to design this study [8]. Semi-structured face-to-face or telephone interviews were conducted with individual community pharmacy stakeholders. Ethical approval was provided by the Research Ethics Committee of the Cardiff School of Pharmacy and Pharmaceutical Sciences before starting the study. Purposive sampling was used to recruit eligible participants [9], and snowball sampling was also used by one participant. Community pharmacy stakeholders who are directly or indirectly involved in commissioning community pharmacy services in Wales were identified using publicly available online contact information and invited to participate in the study via email. Nine invitation emails were sent to identified individuals at the national level (e.g., Welsh Government, LHBs); another general invitation email was also sent to the WPC emailing list. Seven more invitation emails were sent to all research and development offices within the LHBs. For those who were members of CPW and RPSW (members who are directly or indirectly involved in commissioning community pharmacy services), eight and four invitations were sent, respectively. One participant forwarded the general invitation email to other potential participants (five individuals from different LHBs). Of the five, one agreed to take part in the study; however, after sending more than one email to schedule a meeting, the researcher did not receive any further response. Two reminder emails were sent to all other identified potential participants 10 days after the initial contact/first reminder. A Participant Information Sheet was attached to all invitations and reminder emails. Participants were able to choose to carry out the interview face-to-face, via telephone or on Skype. All accessible individuals (i.e., all those who could be identified) who were involved in commissioning/advising on community pharmacy services and agreed to take part were recruited to the study. A topic guide, informed via discussions with the research team and individuals working with a community pharmacy, was developed to explore the future of managing stable chronic conditions in community pharmacy settings (see Supplementary File S1) [10]. Participants were also able to provide any other comments at the end of the interview, should they feel it was necessary. Due to the nature of the topic, the type of participants, and the small population, it was not feasible to pilot the topic guide, but review and reflection after each interview led to the addition of further probes.

Data collection took place between September 2019 and January 2020. Written informed consent was received from participants prior to interview. The interview was designed to take no longer than half an hour. The interviews were audio-recorded via two digital voice recorders (Olympus/VN-732PC). Interviews were transcribed verbatim by a third-party company based in the UK, checked for accuracy by the lead researcher (minor amendments made) and sent to the participant for review and approval. Once saturation was reached (i.e., no new themes emerged), data collection ceased [11].

### Data Analysis

The data were analysed via inductive thematic analysis using NVivo (version 11) software (QSR International Pty Ltd. Melbourne, Australia) [12]. The initial analysis was conducted immediately after generating each transcript, and therefore emergent themes could be identified and included in the topic guide. A six-phase framework was used to analyse the data [12]. At least two authors reviewed the transcripts and the generated themes [13].

## 3. Results

### 3.1. Participants

Ten participants agreed to take part in the study, but one participant did not respond to further communications; therefore, nine interviews were conducted (four face-to-face and five telephone interviews). One participant withdrew from the study after reviewing the generated transcript and their data were not included. The results presented are, therefore, based on the eight remaining participants. The cohort sample was as follows:

- Two participants representing the government and LHBs (henceforth classed as Stakeholders A);
- Five participants representing the Community Pharmacy Wales (classed as Stakeholders B);
- One participant representing the Royal Pharmaceutical Society Wales (classed as Stakeholders B).

### 3.2. Themes

Seven themes were identified from the data set: (1) inconsistency and bureaucracy in commissioning pharmacy services; (2) availability of funding and resources; (3) disagreement and uncertainty about the contribution of the community pharmacy sector; (4) continuity of patient medical information and fragmented care; (5) accessibility, capacity and facilities in community pharmacy; (6) Pharmacy education and clinical expertise, and (7) patient acceptability. There were no differences in the themes identified from face-to-face versus online interviews.

#### 3.2.1. Inconsistency and Bureaucracy in Commissioning Pharmacy Services

It appeared that the current way of commissioning pharmacy services across Wales varied, which caused a lot of tension and disparities within the community pharmacy sector. The lack of consistency in commissioning amongst LHBs was a major issue identified across both Stakeholder groups; further, each LHB had its own requirements and policy to approve and commission pharmacy services. Pharmacy services provided in one LHB may not be available for all community pharmacies in that LHB or even other LHBs; this may lead to unequal opportunities amongst community pharmacies in respect of serving their communities and generating profits, and may also lead to health inequalities among patients, as some of them might not be able to access services that they need. The process of commissioning pharmacy services was described as bureaucratic, involving multiple layers, causing delays in approving services and huge amounts of administrative work.

… *Health boards are a completely different board game, so it depends on the health board. So, you have to understand we come from completely different angles on this (offering pharmacy services)*….

Stakeholder B2

… *I think we’re in a situation currently where pharmacists are looking for more services to do, but health boards in some areas aren’t commissioning them, but in others they are*….

Stakeholder B9

… *I understand there’s inconsistency because sometimes health boards take on the role of assurance in a way that conflicts with other bodies who are already giving assurance; I think that is an area that needs to be worked on*….

Stakeholder A4

… *We have cases of health boards not liking the answers for what’s agreed at a national level and therefore, not implementing it even though it may be implemented in the other six health boards*….

Stakeholder B5

… *I think in the main, there are delays, the process is very unwieldy for commissioning. There are many, many, many people involved*….

Stakeholder A6

#### 3.2.2. Availability of Funding and Resources

Almost all participants identified that providing a community-based stable chronic condition management service might not be feasible without additional funding and a change in the remuneration model to reflect the new direction of travel may be needed. The additional funding might be spent on hiring new staff, training, purchasing tools and equipment, and incentivising pharmacy staff. The current community pharmacy funding model focuses on dispensing; it is a quantity-driven model, which might be inappropriately used to generate revenue regardless of the meaningfulness of the intervention. In recognising that management of stable chronic conditions may require extended/repeat consultations with individual patients, the remuneration model should focus more on the ‘quality’ of care provided.

… *The remuneration model for dispensing is to chase as many items as you can possibly do, dispense them as quickly as you possibly can and give them out as quickly as you possibly can [laughs]. There’s no…no focus on quality, it’s on volume*….

Stakeholder B3

… *So MURs, it’s all about volume, it’s not about quality. It doesn’t matter if you spot a problem or not, doesn’t matter if they’re on two medicines or 20, doesn’t matter if they’re frail, elderly or, or 50-year-old. You’re gonna do the simple patients on the minimum medicines, because that is the revenue generator*….

Stakeholder A6

… *The barrier that comes hot on the tail of that is how do you create enough time and space and resource to train the workforce to do that*….

Stakeholder A4

… *I think we are embarking on a period of shortage within the pharmacy, community phar-, community pharmacy is struggling to recruit in many areas. I think we’re probably bordering on a, a bit of a workforce crisis*….

Stakeholder B8

#### 3.2.3. Disagreement and Uncertainty about Contribution of the Community Pharmacy Sector

Participants felt that a proportion of contractors and community pharmacists might have different perspectives from the Welsh Government about the contributions of community pharmacy to people’s health; they might not be in favour of moving away from dispensing towards providing more clinical services. How community pharmacy is reimbursed might be one of the reasons, but there might be other factors, too. Experienced pharmacists would perhaps be less interested in providing clinical services; indeed, they might be more comfortable doing what they are currently doing. As community pharmacies are run by contractors, the Welsh Government might face difficulties in achieving their vision regarding the community pharmacy sector. Community pharmacy as a sector should fully understand its new orientation and why it is moving in that direction; further, the direction of travel and the future contribution of community pharmacy to people’s health might not be fully understood by community pharmacists. The community pharmacy sector is currently in a transition stage, a stage where a significant change in its vision and contributions is planned to occur in the future. It appears that better communication is needed between community pharmacies and their regulators.

… *The pharmaceutical bodies and the, the government and other people, in my opinion, do not fully value the dispensing process, and they see it as a commodity-based service*….

Stakeholder B7

… *I think the final challenge is a need for a change in the mindset of the sector, so individual pharmacists get this, some pharmacy contractors get it, but equally, some pharmacists and some pharmacy contractors don’t: they’re quite happy to put their fingers in their ears and say actually, I want to keep doing what I’ve always done and I don’t want to expand what I do; and even if we make the incentives right and make the disincentives right, there will still be people who don’t want to do it*….

Stakeholder A4

… *The comments of Welsh Government talk about a changing role, you know… replacing supply with clinical services, but we haven’t replaced it, we’ve added it on. At the moment, nothing has gone*….

Stakeholder B3

… *I think really importantly, for the sector, it needs to change what it does, so I make a big point of trying to explain to community pharmacists that it’s not… a drive to move away from dispensing, is not… the government isn’t necessarily forcing that to happen, consumer behaviour is the thing that will drive that*….

Stakeholder A4

#### 3.2.4. Continuity of Patient Medical Information and Fragmented Care

Most of the participants identified a critical issue in the primary care setting in respect of continuity of information among different healthcare settings. Community pharmacy was not well connected with other primary healthcare settings. When patients move from one primary setting to another, not only should they move seamlessly, but also all relevant information should move as well. The places where primary care services are provided should be joined up, allowing for a continuation of care when patients move from one place to another. Having one medical record for each patient that could be accessed by different practitioners across different healthcare settings may help in enhancing the care provided.

… *So even though we’ve got fabulous services over here, the poor services over here are what people talk about. They don’t talk about where services are working, ‘cos often the GPs don’t even know that their patients are accessing a service (a service which was provided in a community pharmacy), because that’s working*….

Stakeholder A6

... *Every pharmacist will need access to patient records, really. Without full access to patient notes, that position (managing stable chronic conditions) becomes extremely difficult*….

Stakeholder B9

#### 3.2.5. Accessibility, Capacity, and Facilities in Community Pharmacy

There was general agreement amongst participants that community pharmacy had excellent accessibility; this accessibility of the community pharmacy was not only related to being available within short distances from where people live, but also being accessible for longer hours and on days when no other primary care settings are open. Given that stable chronic conditions are more frequent in elderly people, offering a stable chronic condition management service in their local pharmacy might be more convenient for them; further, this would allow them to be managed and receive their medications in a single place.

*… Most people are within a very short journey of their pharmacy*….

Stakeholder B3

… *Because they are often open longer hours for the retail offer, which then means that there’s better access*….

Stakeholder A6

Almost all participants agreed that it would be difficult to manage stable chronic conditions in a community pharmacy with the current type of practice. Community pharmacists had heavy workloads, making it unfeasible to expand their role in managing stable chronic conditions in the absence of other changes. Managing stable chronic conditions properly would require community pharmacists to spend more time looking at patient medical records and counselling patients. All of this would add to their workload, and might hinder their ability to complete other tasks assigned to them. The quality of service provided might be affected and the opportunity for medication errors to occur might increase as the workload increases.

… *If we don’t release some capacity to make some headroom for community pharmacists to evolve that role, there’s just not the space there to do at the moment*….

Stakeholder B8

… *I don’t think it’s feasible (managing stable chronic conditions in pharmacy) with current models of work. We’re—I think 75 million prescription items are supplied through community pharmacy in Wales each year*….

Stakeholder A6

… *If you’ve got a technician doing your supervised consumption and smoking cessation service, you need a second consultation room, maybe a third. Many community pharmacy premises are not fit for purpose*….

Stakeholder A6

Community pharmacy settings may require further improvement prior to managing people with stable chronic conditions.

… *Most of our consulting rooms now are more on a…. a face to face consultation over a desk rather than an examination couch*….

Stakeholder B3

#### 3.2.6. Pharmacy Education and Clinical Expertise

Community pharmacists represent a large group of healthcare professionals in Wales; their current contributions to patient care might go beyond their current role. Tasks that could be performed by other pharmacy staff (e.g., pharmacy technicians) should not be assigned to the community pharmacist. Community pharmacists should be assigned tasks in which they add value.

… *I don’t think we’ve got as much of a role (in managing stable chronic conditions) as we could have*….

Stakeholder B3

… *We have got an untapped resource in terms of the skills and the rapport and the trust that sits in a community pharmacy. We’re not using it very effectively. We’re—we’ve got a big sports car and we’re driving it round at 20 mile an hour everywhere*….

Stakeholder A6

Whilst almost all participants were in favour of expanding the role of community pharmacists in managing stable chronic conditions, they were concerned about the clinical expertise of pharmacists. Managing stable chronic conditions is not about being an expert in all aspects of medicines. Community pharmacists would need to use advanced clinical skills that would enable them to manage patients properly (e.g., identifying disease progression, and interpreting test results). The current pharmacy education and pre-foundation programs might not be primarily focused on the clinical side. To be able to manage stable chronic conditions properly, community pharmacists may need to expand their knowledge and enhance their clinical skills, not to mention that most practising community pharmacists qualified many years ago (i.e., their pharmacy education programs might not include as much clinical information as the current ones).

… *The bulk of our workforce in community pharmacy are 10, 15, 20, 25 years qualified. They qualified from an MPharm degree or a BSc degree, BPharm degree, that didn’t include a lot of clinical information, or nothing like it is now*….

Stakeholder A6

… *I think there’s a couple of areas that we would need to improve on, and partly that’s to do with record writing, and partly that’s to do with some of the more examinational skills*….

Stakeholder B5

As current community pharmacists’ clinical knowledge and expertise in managing stable chronic conditions might be insufficient, patient health might be affected; they might make inappropriate medical decisions that put patients’ lives at risk. The LHBs, employers and community pharmacists should all be clear about where liability falls if patients are harmed.

… *Somebody will get hurt, yeah, from a consultation, there will be a fatality, there’ll be sepsis missed. That has already happened with a paramedic*….

Stakeholder B2

#### 3.2.7. Patient Acceptability

Patients would be the beneficiaries of stable chronic condition management services; their willingness to have their stable chronic condition managed by a community pharmacist is an important enabler to having a successful model. Several participants indicated that patients might be reluctant to have stable chronic health conditions managed by a pharmacist; this might be attributed to the historical stereotype about community pharmacists being dispensers. Patient perceptions about community pharmacies need to reflect the new direction of travel; they should see community pharmacists as “healthcare providers”, not as “dispensers”, and community pharmacies as “health and wellbeing hubs”, not as “places to pick up prescriptions”.

… *So, I think patients’ perceptions about where they get these services [managing stable chronic conditions] done needs to change, and that could risk our ability to do that*….

Stakeholder B5

… *Some patients wouldn’t want to change, so… and this is coming back to my point of trying to be a mix of services, so those patients who actually value being seen by their consultant or seen by their GP, we don’t want to remove those options from people, we want to increase choice and accessibility, not change choice and accessibility*….

Stakeholder A4

## 4. Discussion

The results of the present study identified seven themes related to managing stable chronic conditions in community pharmacy settings. These limitations might hinder the expansion of community pharmacy’s role in managing stable chronic conditions. Further consideration would be needed prior to enacting the plan to increase the involvement of community pharmacists in the clinical management of stable chronic conditions.

The findings from this study demonstrate that there is potential for increased clinical services to be run from community pharmacies. Community pharmacists could contribute more to people’s health and wellbeing. Several studies have shown that interventions conducted by community pharmacists improved patient care outcomes in multiple stable chronic conditions, such as diabetes, hypertension, and cardiovascular diseases [14,15,16]. More importantly, randomised clinical trials have shown that interventions conducted by pharmacists reduced inappropriate prescribing, side effects, medication use, and spending on medicines [17,18]; however, the current involvement of community pharmacists in dealing with stable chronic conditions is limited to a few services, such as Medicine Use Review (MUR) and Discharge Medicine Review (DMR). Even though these services might have some benefits for patients, the services are limited in scope. It was argued that the way in which the MUR service was constructed (i.e., focused mainly on medicine itself rather than on the underlying condition(s)) may not really improve patient outcomes [19,20].

Involving community pharmacists in managing stable chronic conditions would potentially have several advantages at multiple levels (i.e., individual, community pharmacy, and healthcare structure). Firstly, it may improve job satisfaction and reduce the number of pharmacists leaving the sector. Community pharmacists have lower job satisfaction in comparison to hospital pharmacists, which might lead to more leaving the profession [21,22]. Secondly, involving community pharmacies in managing stable chronic conditions would make pharmacy businesses more sustainable and profitable; it would make it difficult to replace community pharmacies with technology, as the focus would be shifted from dispensing to providing direct patient care. Thirdly, it would improve health inequality, as patients would be able to access healthcare services at their convenience, in places where access to GP or secondary care services is currently limited. Patients could easily access a community pharmacy within a few minutes’ walk from where they live [23]. More importantly, pharmacies are more accessible in the most deprived areas, which are where stable chronic conditions are prevalent [24]. Fourthly, involving pharmacists in managing stable chronic conditions might also reduce spending on health, as detecting health issues earlier and providing better care might contribute to less morbidity [25,26]. Finally, it would also reduce the burden on GPs freeing up capacity, which is currently under immense pressure [3]. All of these factors could improve patient care, accessibility to health services, job satisfaction, business sustainability, and decrease the burden on the NHS and GP services.

The community pharmacy sector has been through a range of fundamental changes toward providing more direct patient care, which has helped in freeing up GPs’ time and providing convenient health services for patients [7,27,28]. As identified in this study, the planned transformation in community pharmacy might not yet be fully understood by people involved in community pharmacy settings (e.g., pharmacists and contractors). It is fundamental that community pharmacies understand the importance of changes and the direction of travel (i.e., moving towards providing more clinical services in pharmacy). Although changing the contractual framework might help in encouraging some practices, getting people involved in community pharmacy who are supportive of the changes may assure that they deliver the service for its own sake. Achieving the goals set for the community pharmacy sector will require collaboration from all involved. Effective communication with contractors, explaining the need for changes, may help to conceptualise the problem. Providing equal opportunities for all contractors to generate revenue from other pharmacy services rather than dispensing may be helpful. Additionally, providing necessary training to provide the new clinical services, and supporting pharmacists to work collaboratively in a multidisciplinary team might all help in this regard.

One of the biggest issues expressed by participants that threatens the goal of managing stable chronic conditions in community pharmacies is the way LHBs commission pharmacy services. There are seven LHBs regulating community pharmacies in Wales [29,30]. Each LHB determines which services can be provided in each community pharmacy within the Board based on the needs of the community [30]; this might impact health equality and the opportunity for contractors to improve their communities and generate enhanced revenues. Even when a service has been approved at a national level (i.e., National Enhanced Services Board), participants claimed that LHBs may change or add additional requirements; this may make approving a service at the national level less helpful. There should be an equal opportunity for all contractors to serve their community and also to generate sustainable revenues. Currently, the main revenue generator for community pharmacies is dispensing [31]. All community pharmacies are required to provide this service [32]. Therefore, the opportunity to dispense medications is equal for all contractors because it has been approved as an essential service; this provides community pharmacies with an equal opportunity to generate profits, and in the meantime prevents variations in service commissioning. More importantly, it ensures that a large proportion of the community pharmacy fund is distributed fairly among community pharmacies; however, the funds allocated for dispensing have been reduced over the years and more funds have been allocated to provide more clinical pharmacy services [28,33,34]. This means that the revenue generated by a community pharmacy is likely to be significantly impacted by a decision regarding the provision of a service taken at an LHB level. A community-based stable chronic condition management service should therefore be approved at a national level, without allowing LHBs to add extra requirements, such that any qualified community pharmacist willing to provide the service and meeting the standardised service requirements can provide it.

Our study has highlighted that there are concerns that the community pharmacy sector may not currently be ready to manage stable chronic conditions. Further improvements/facilitators might be needed at the national level (policy and regulations that govern the community pharmacy sector), community pharmacy level (capacity and competencies within community pharmacies) and patient-level (acceptability to patients of having their conditions managed in community pharmacy). Commissioners of community pharmacy sectors will likely have to implement legislation that reduces disparities among LHBs, ensures patients’ safety and integrated care, as well as provides financial sustainability for the service. More work is needed to prepare the community pharmacy sector for meeting the anticipated requirements of managing stable chronic conditions; this would likely include preparing community pharmacists to be independent prescribers and ensuring that their premises are suitable for managing people with stable chronic conditions such as providing adequate consultation room capacity. Increasing awareness of the public about the role of community pharmacists as “healthcare providers” and the value that they could add to improve patients’ health outcomes is also needed.

To achieve the vision for community pharmacy, making community pharmacy a hub for improving people’s health and wellbeing, the WG introduced new legislation and made changes to the contractual framework [34]. The contractual changes should encourage community pharmacists to provide clinical services as the funds allocated for dispensing medications are decreasing annually. The workload distribution in community pharmacies will change as community pharmacy teams will be assigned new tasks, allowing pharmacists to contribute more to people’s health by the provision of clinical services; in addition to this, pharmacy technicians will be better utilised to take on tasks that are currently performed by pharmacists. To reduce disparities among LHBs with respect to commissioning clinical services, the WG plans to introduce four priority services as national ones; these services will have specific standards, and therefore any community pharmacist that meets these standards will be able to provide the service across Wales. These changes in the contractual framework will empower community pharmacies to play a crucial role in people’s health and wellbeing and ensure the sustainability of community pharmacies.

### Strengths and Limitations

The present study was the first to explore the views of community pharmacy stakeholders in Wales; it involved participants who were involved in the community pharmacy sector from the government, LHBs, CPW, and RPSW. Some participants were also members of other pharmaceutical groups/boards; this allowed exploration of the research topic from different perspectives and backgrounds. Using in-depth interviews allowed for a deep understanding of the topic. Nevertheless, there were a few limitations to the present study. Due to difficulties in conducting face-to-face interviews, some interviews were conducted via telephone; it was thus not possible to observe non-verbal communication, and furthermore, due to technical issues, the quality of the calls was sometimes affected. For instance, phone calls were sometimes cut off/reception was lost, partially impacting the quality of the generated transcripts; however, the researcher ensured that the collected information was accurate by repeating questions and asking for clarification during the interviews. Furthermore, the participants had the chance to review and approve the transcripts, which should improve the quality of the collected information. Lastly, the focus of the current study was on stakeholders directly or indirectly involved in the commissioning of community pharmacy services. Additional important insights may be identified from studies where the views of patients, GPs and community pharmacists themselves are surveyed.

## 5. Conclusions

Whilst there are many potential benefits to more in-depth management of stable chronic conditions in community pharmacy, including the accessibility of community pharmacies and the expertise of the pharmacists working in the sector, the stakeholders in this study recognised the need for enhanced training, resources, and support before the expansion of services could take place. It is therefore important for commissioners of pharmacy services to lay the foundations now so that pharmacists and patients alike can make the most of this important opportunity to support patients with stable chronic conditions to better outcomes.

## Data Availability

Data are available upon request from the corresponding author. The data from this study are not publicly available due to the potential that participants might be identified from the transcripts.

## Supplementary Materials

The following supporting information can be downloaded at: https://www.mdpi.com/article/10.3390/pharmacy10030059/s1, File S1: Topic guide.
