# Supplementary material for: Stakeholders’ Views about the Management of Stable Chronic Conditions in Community Pharmacies
Source: Pharmacy (Basel). 2022 Jun 2;10(3):59. doi: 10.3390/pharmacy10030059 (PMC9231151; doi:10.3390/pharmacy10030059)
Supplement: Supplementary file 1 [file pharmacy-10-00059-s001.zip › pharmacy-1752353-supplementary.pdf]

pharmacy MDPI

Supplementary File S1: Topic guide

Cardiff University Prifysgol Caerdydd

Cardiff School of Pharmacy & Pharmaceutical Sciences

Commissioners of pharmacy services views about the management of chronic disease in community pharmacies

Interview Schedule and Script

Introduction:

Hello,

- Greeting and welcoming
- Introduce my self
- Aim of the project
- Reminder that the interview is recorded

1. Do you have any questions?

Let's get started!

Current practice: 1- What do you think of contributions of community pharmacists to the current mission of NHS? *Prompt: their tasks as a community pharmacist on a daily basis, services provided to the patients*

Prompt1: you mentioned community pharmacists providing …………… to the patients, How do you feel about type of care provided for patients in community pharmacy? Prompt: Such as, clinical review services, smoking cessation, weight management.

Prompt2: What do you think needs to be changed in the future? If they mentioned something ……What are the drivers of these changes? Number of GPs, waiting time, access to health services.

Transition: (based on participants' answered) "you said community pharmacy should be involved (effectively utilized) in clinical services provided to the patients…………….! (link to the next section)

Proposed plan (managing chronic diseases): 2- What is the future of community pharmacy involvement in managing chronic diseases?

Prompt1: How do you see feasibility of community pharmacists being managing people with chronic diseases in terms of capacity, capability and funding.

Pros and cons: 3- What are the pros and cons of managing people with chronic diseases in community pharmacy? *Prompt: Impact on patient care, health system (GPs), other health professionals, money allocation, number and distribution of community pharmacies/pharmacists*

Prompt1: you mentioned ……………. that might hinder expanding of pharmacy services, what are other limitations of expanding the role of community pharmacists in managing patients with chronic diseases? Prompt: personal, organizational, policy, funding.

Prompt2: How could these barriers be overcome?

Closing:

That's all what we have, thank you again for your participation in this conversation. Do you want to add anything to our discussion?

A list of general prompt questions that might help during the discussion:

Could you please clarify……?

Can you explain a bit more about that?

You slightly covered this point …………… could you elaborate a bit on …………..?
